# Enzymatic Fluoromethylation as a Tool for ATP‐Independent Ligation

**DOI:** 10.1002/ange.202312104

**Published:** 2023-11-29

**Authors:** Jiaming Peng, Gregory R. Hughes, Manuel M. Müller, Florian P. Seebeck

**Affiliations:** ^1^ Department of Chemistry University of Basel Mattenstrasse 24a 4002 Basel Switzerland; ^2^ Department of Chemistry King's College London Britannia House 7 Trinity Street SE1 1DB London UK

**Keywords:** Fluorine Biocatalysis, Methyltransferase Biocatalysis, Native Chemical Ligation, Post Translational Modification, Protein Synthesis

## Abstract

*S*‐adenosylmethionine‐dependent methyltransferases are involved in countless biological processes, including signal transduction, epigenetics, natural product biosynthesis, and detoxification. Only a handful of carboxylate methyltransferases have evolved to participate in amide bond formation. In this report we show that enzyme‐catalyzed F‐methylation of carboxylate substrates produces F‐methyl esters that readily react with *N*‐ or *S*‐nucleophiles under physiological conditions. We demonstrate the applicability of this approach to the synthesis of small amides, hydroxamates, and thioesters, as well as to site‐specific protein modification and native chemical ligation.

## Graphical abstract

This report shows that methyltransferase‐mediated fluoromethylation can activate small and large carboxylate substrates for conjugation to thiols, hydrazine, hydroxylamine or amines. This methodology to produce anhydrides offers an alternative to the much more common ATP‐dependent processes observed in nature and utilized in biocatalysis.

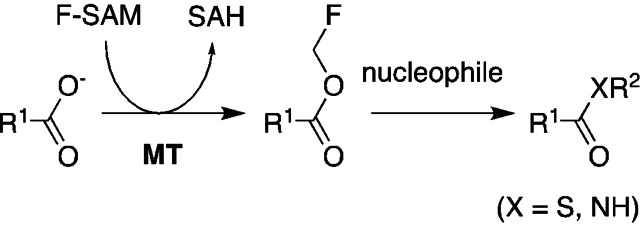

Amide bond formation is one of the most important reactions in biology and also in synthetic chemistry, since amides occur in many therapeutics, agrochemicals, food additives, plastics, and dyes. Consequently, a large body of chemical literature documents methodologies, reagents and processes to connect amines with carboxylates, continuously expanding the scope, efficiency, selectivity, and economic viability of condensation reactions.^[1]^ Biocatalysis plays an increasing role in this development, empowered by the growing list of known amide bond forming enzymes and increasing ability to engineer enzymes with tailormade amide bond forming activities.^[2]^ Under physiological conditions, the central challenge to condensation reactions between amines and carboxylates is that the reaction equilibrium strongly favors the educts. The same problem applies to the formation of other anhydrides including esters, thioesters, and hydroxamates, with notable exceptions such as disulfides and thioethers.

In cellular metabolism, most condensation reactions are driven against this equilibrium using adenosine triphosphates (ATP and other nucleoside NTPs) as an energy source.^[2d]^ ATP‐dependent ligases conjugate their carboxylate substrates with either phosphate or adenosine‐5′‐monophosphat (**A**, Figure 1). Depending on the involved enzymes, these anhydrides can react with amine nucleophiles to form amides, or may be transferred to carrier molecules such as the 3′‐hydroxyl function of transfer RNAs, or the thiol function of Coenzyme A (CoA) or of a 4′‐phosphopantetheine moiety of an acyl carrier protein.^[2b]^ Enzyme‐catalyzed ATP‐dependent amide bond formation has been exploited successfully in preparative reactions (Figure 1).[^2d^, ^3^] The most promising examples include an ATP‐recycling cascade that uses polyphosphates instead of ATP as the stoichiometric dehydration agent.^[4]^

**Figure 1:**
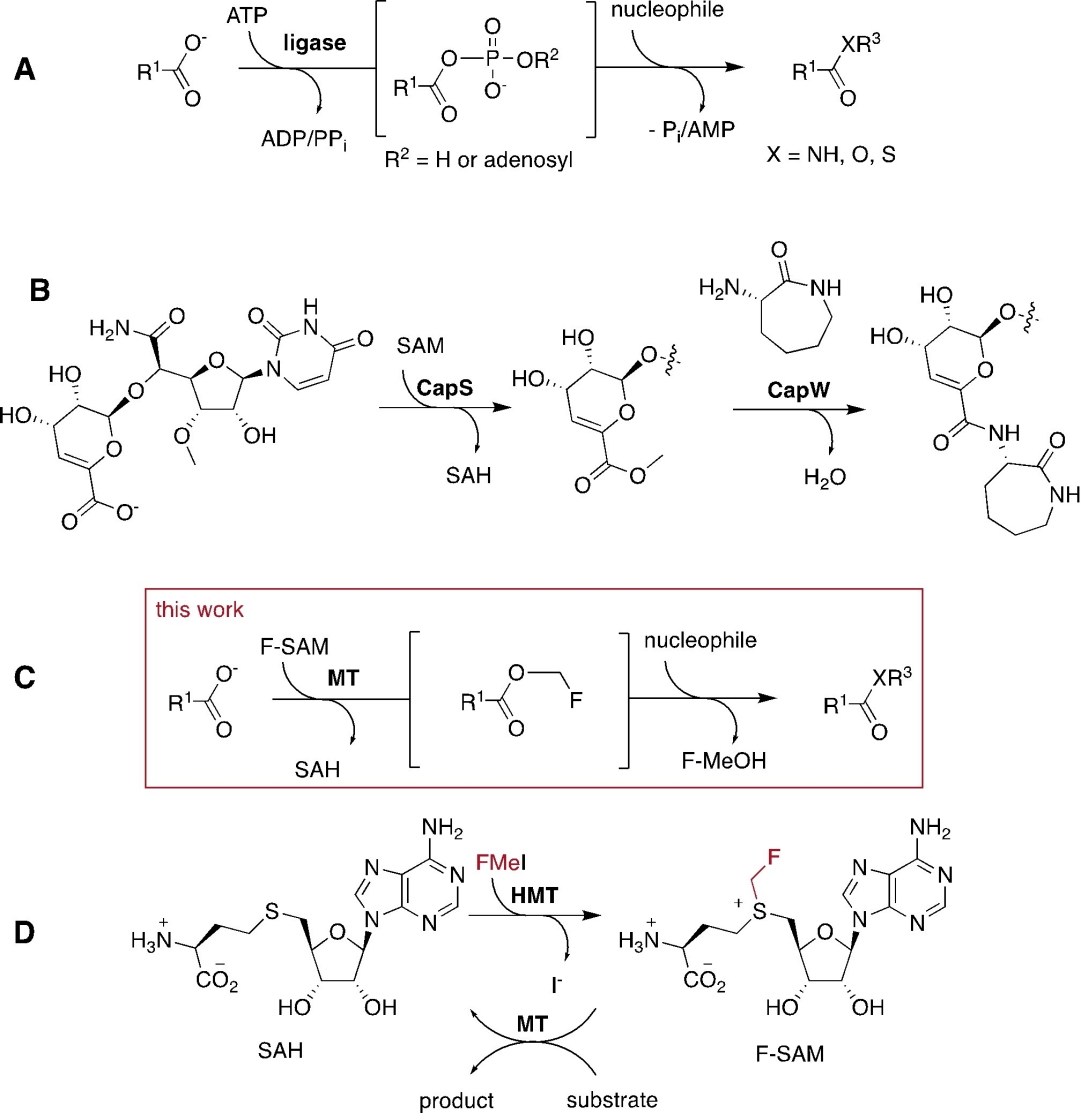
**A**: ATP‐dependent ligases activate carboxylate substrates by producing phosphate or adenosine‐5′‐monophosphat (AMP) esters. **B**: Specific carboxylate MTs (CapS) combined with matching acetyltransferases can mediate amide bond formation by a SAM‐dependent mechanism. **C**: Carboxylate MT‐catalyzed F‐methylation provides a more broadly applicable mechanism for ATP‐independent anhydride‐formation. **D**: HMT‐catalyzed production of F‐SAM as a reagent for carboxylate F‐methylation.

There are surprisingly few documented enzymes that activate carboxylates without ATP or other NTPs. A rare example participates in the biosynthesis of the nucleoside antibiotic capuramycin (**B**, Figure 1). In this process a *S*‐adenosyl methionine (SAM)‐dependent methyltransferase (CapS) methylates the carboxylate of a biosynthetic intermediate.^[5]^ The resulting methyl ester is conjugated with the amine nucleophile of aminocaprolactam by the acyltransferase CapW. The discovery of this pathway led to the realization that carboxylate methyltransferases (MTs) may be used in ATP‐independent ligation reaction—provided that acyltransferases with matching substrate scope can be identified.[^2e^, ^6^]

This present report is inspired by this idea. We describe an MT‐catalyzed process for the formation of hydroxamates, thioesters and amides without the need of ATP or acyltransferases. We have harnessed this methodology for the synthesis of low molecular weight hydroxamates, thioesters and amides, for specific labeling of iso‐Aspartate (isoAsp) containing peptides, for C‐terminal protein labeling, and protein‐protein ligation.

This methodology builds upon our earlier discovery that SAM‐dependent halide methyltransferase (HMT) can transfer fluoromethyl groups (F‐methyl) onto *S*‐adenosylhomocysteine (SAH) to generate fluorinated SAM (F‐SAM, Figure 1).^[7]^ Carboxylate MTs can transfer the F‐methyl group from F‐SAM to their cognate substrates. Importantly, the resulting F‐methyl esters are significantly more reactive than the corresponding methyl esters. For example, the trans‐aconitate 3‐methyltransferase from *Saccharomyces cerevisiae* (TAMT, EC 2.1.1.145, PDB code: 3G5T) F‐methylates 3‐isopropylmalate (**1**, Figure 2) to form the F‐methyl ester **2 a**.^[7]^ In the presence of 5 mM hydroxylamine the accumulating product is isopropyl malic hydroxamic acid (**3**). Similarly, F‐methylation of **1** in the presence of L‐cysteine (4 mM), fluoromethyl iodide (FMeI, 4 mM), HMT and TAMT (both 10 μM) at pH 8.0 in 50 mM phosphate buffer resulted in complete conversion of 1 mM **1** to the cysteine conjugate **4** after 19 hrs at 25 °C as inferred by HR‐ESI‐MS (Figure 2, [C_10_H_16_NO_6_S]^−^ calcd: m/z 278.0703, found: 278.0707, Figure S1). In contrast, a reaction containing MeI instead of FMeI produced 3‐isopropylmalate methyl ester (**2 b**, [C_8_H_13_O_5_]^−^ calcd: m/z 278.0703, found: 278.0705, Figure S2), but no detectable cysteine conjugate **4**. Reactions lacking either HMT or TAMT produced neither ester nor amides, consistent with the expectation that both MeI and FMeI are comparatively poor reagents for uncatalyzed methylation of carboxylates.

**Figure 2:**
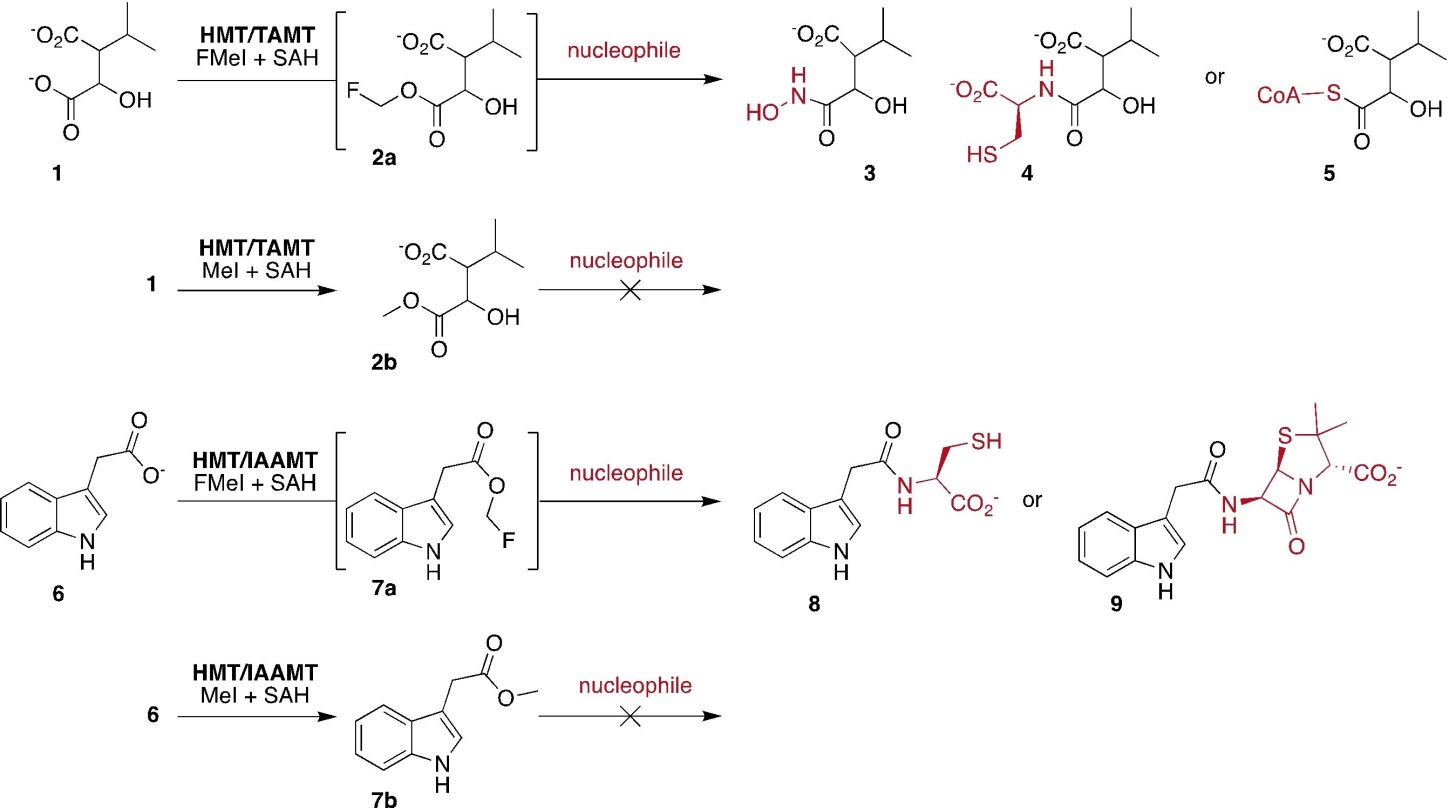
MT‐catalyzed F‐methylation activates low molecular weight carboxylates for uncatalyzed formation of hydroxamates, amides and thioethers. The methyl esters of the same carboxylates react much less efficiently with nucleophiles.

We produced compound **4** in a larger scale reaction (64 μmol of **1**, 108 μmol of Cys) and alkylated the thiol of **4** by treatment with 4‐bromomethyl‐7‐methoxy‐coumarin. The resulting conjugate **4 a** could be purified by semi‐preparative reversed phase HPLC (Scheme 1). This procedure produced compound **4 a** from **1** with 78 % isolated yield (Figure S3–S5). The structure of **4 a** was confirmed by ^1^H and ^13^C NMR (Figure S6–S10). F‐methylation in the presence of 2 mM coenzyme A (CoA) converted **1** (2 mM) to the corresponding thioester **5** ([C_28_H_47_N_7_O_20_P_3_S]^+^ calcd: m/z 926.1804, found: 926.1802, Figure S12) within 30 min as inferred by HPLC (Figure S11). Again, no conjugation was observed in a reaction containing MeI instead of FMeI (Figure S13).

**Scheme 1:**
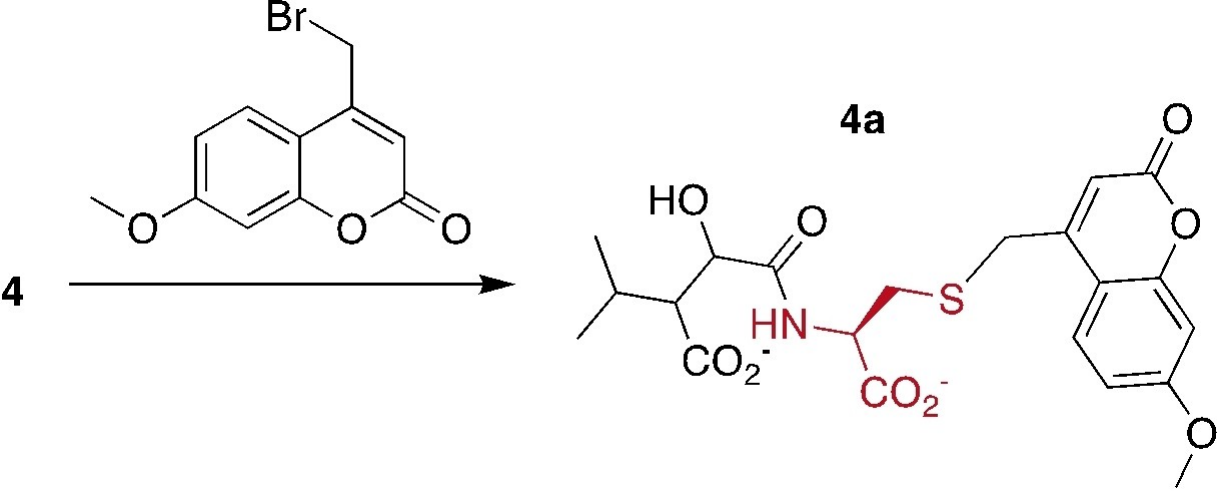
Chemical alkylation of **4** with 4‐bromomethyl‐7‐methoxy‐coumarin.

To examine a second example of a small molecule carboxylate MT we produced the indole‐3‐acetic acid MT (IAAMT, EC:2.1.1.278, PDB: 3B5I) from *Arabidopsis thaliana*.^[8]^ A reaction containing IAAMT and HMT (both 20 μM), 4 mM FMeI, 40 μM SAH and 4 mM Cys converted 1 mM indole‐3‐acetic acid (IAA, **6**) to the corresponding cysteine conjugate (**8**, [C_13_H_15_N_2_O_3_S]^+^ calcd: m/z 279.0798, found: 279.0799, Figure S14). HPLC analysis showed that this reaction is competed after 18 h. (Figure S15). In a control reaction without Cys, we observed the transient formation of IAA F‐methyl ester (**7 a**, [C_11_H_11_FO_2_N]^+^ calcd m/z: 208.0768, found 208.0771, Figure S16), but no irreversible consumption of the substrate. (Figure S17). A reaction containing MeI in place of FMeI produced IAA methyl ester (**7 b**, [C_11_H_12_O_2_N]^+^ calcd m/z: 190.0863, found 190.0863, Figure S19) but no cysteine conjugate **8** (Figure S18). In addition to esterification, we also observed that MeI methylated the thiol side chain of cysteine ([C_4_H_10_O_2_NS]^+^ calcd m/z: 136.0427, found 136.0427, Figure S19).

Conjugation of Cys to **1** and **6** likely proceeds via nucleophilic attack of the Cys thiolate onto the F‐methyl ester, followed by *S*‐to‐*N* acyl transfer.^[9]^ To test as to whether F‐methyl esters would also react with amines directly, we examined the products of the same IAAMT and HMT catalyzed reaction, containing 10 mM 6‐aminopenicillanic acid instead of Cys (Figure 2). HPLC analysis showed that this reaction converted 80 % of **6** to the corresponding conjugate **9** ([C_11_H_23_N_2_O_4_]^+^ calcd: m/z 374.1169, found: 374.1175, Figure S21) within 21 h (Figure S20). 20 % of **6** remained unreacted. These results demonstrate that a thioester intermediate is not required for conjugation, and that comparatively bulky nucleophiles are tolerated.

**Peptide and protein labeling**: Encouraged by these results with small carboxylates we explored as to whether MTs could also mediate conjugation to peptides or proteins. First, we studied the example of protein‐L‐isoaspartate O‐methyltransferase (PIMT). This enzyme methylates the α‐carboxylate of isoAsp moieties in proteins and peptides that underwent spontaneous isomerization of the peptide backbone (**10**, Figure 3).^[10]^ The resulting isoAsp methyl ester (**11**) reacts to a succinimide intermediate (**12**) that then hydrolyzes either to reproduce the starting isoAsp moiety, or an Asp residue with an eupeptide bond (**13**). The former reaction returning to **10** occurs approximately 3‐fold faster than the latter reaction.[^10b^, ^11^] Hence, multiple rounds of methylation by PIMT, cyclization and hydrolysis may be necessary to restore the activity and stability of damaged protein. At the present time, the physiological function of this activity in animals, plants, fungi and bacteria is not clear.[^12^, ^13^, ^14^] In order to address this question, a number of reports describe labeling techniques to identify and quantify potential PIMT substrates.[^15^, ^16^]

**Figure 3:**
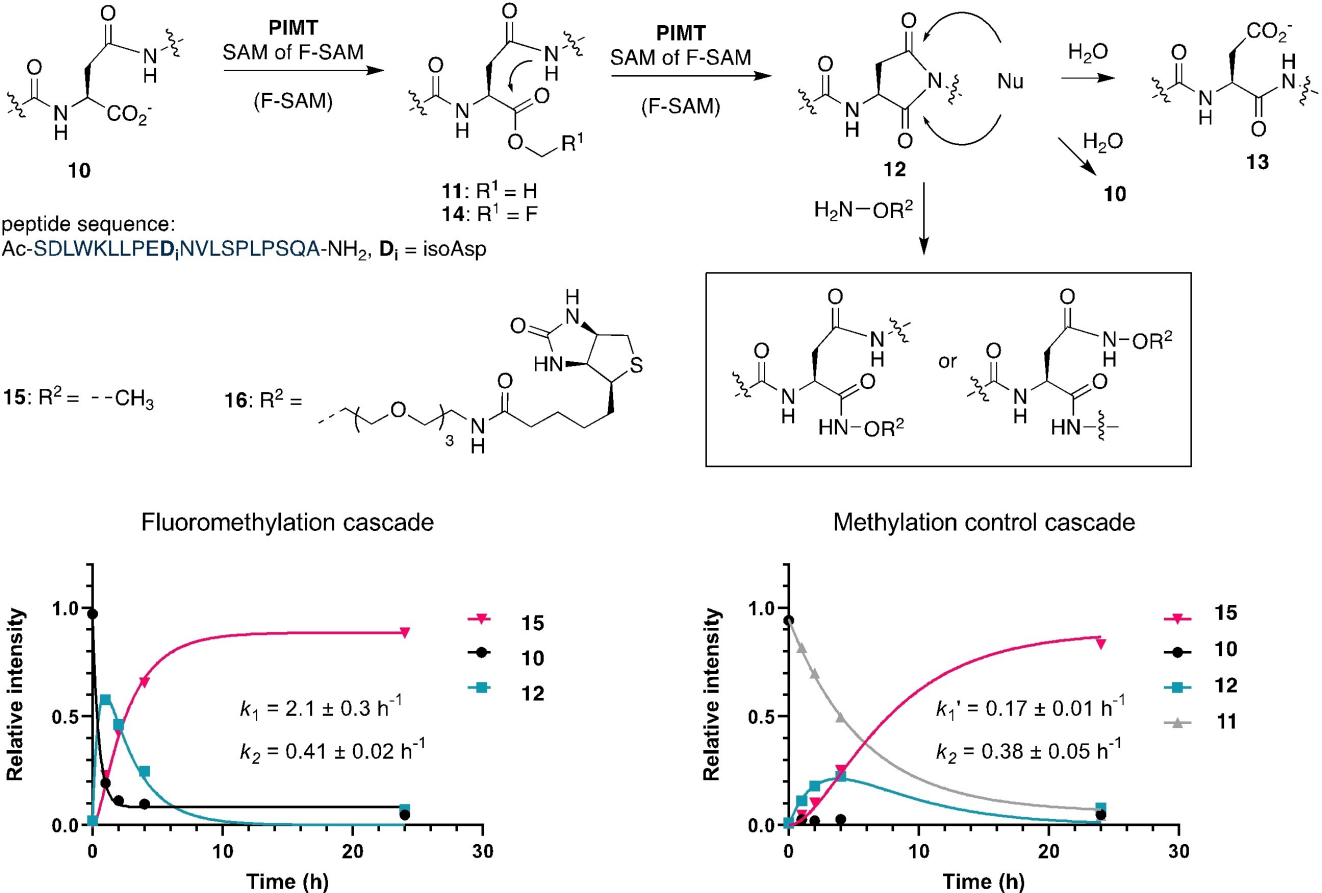
PIMT‐mediated peptide labeling. **Top**: PIMT‐catalyzed methylation or F‐methylation of isoAsp moieties in a model peptide (**10**) produces the corresponding methyl ester (**11**) or F‐methyl ester (**14**). Both esters collapse to a succinimide intermediate (**12**) that can react with an external nucleophile to form an O‐methyl hydroxamic acid (**15**). **Bottom**: Reaction progress of nucleophilic trapping of an isoAsp‐containing peptide upon PIMT‐dependent F‐methylation (left) and methylation (right). The reactions are fit to a 2‐step sequential kinetic model as described in the SI. The ranges correspond to the standard error of the fit. The constants *k*_1_ and *k*_1_′describe the rate at which intermediate **12** is formed; *k*_2_ describes the rate at which intermediate **12** is conjugated to methoxyamine.

One type of technique is based on the idea that PIMT‐treated proteins could be trapped with a high concentration (>1 M) of synthetic nucleophile such as hydrazine or Tris (2‐Amino‐2‐(hydroxymethyl)‐1,3‐propanediol) to facilitate enrichment and identification of conjugated PIMT substrates.[^15a^, ^15c^, ^16^, ^17^] This Scheme is elegant, but the protocols published so far are not efficient enough to detect isoAsp‐ or succinimide‐containing peptides in complex samples. Hence, improving the efficiency of isoAsp‐conjugation reactions is an important objective.

To test as to whether F‐methylation could help in this regard, we incubated a synthetic isoAsp‐containing peptide (**10**, [C_103_H_165_N_25_O_32_]^2+^ calcd: m/z 1132.604, found: 1132.595, Figure 3 and S22) with 50 μM HMT and 2.8 μM human PIMT (PDB: 1I1N), 5 mM FMeI or MeI and SAH (50 μM) in 0.1 M phosphate buffer at pH 7 in the presence of 1 M methoxyamine. The progress of this reaction was monitored by liquid chromatography‐coupled MS (Quadrupole Time of Flight Mass Spectrometer) and the signal intensities for different peptide species were used to estimate their relative concentration (Figure 3). The FMeI containing reaction afforded almost complete conversion of substrate peptide to the succinimide‐containing derivative within the first hr (**12**, ([C_103_H_163_N_25_O_31_]^2+^ calcd: m/z 1123.598, found: 1123.590, Figure S22). The intermediary F‐methyl ester (**14**, [C_104_H_166_FN_25_O_32_]^2+^ calcd: m/z 1148.607) did not accumulate under these conditions. After four hrs, most peptide was converted to the O‐methyl hydroxamic acid (**15**, [C_104_H_168_N_26_O_32_]^+^ calcd: m/z 1147.117, found: 1147.106). Since the intermediate succinimide **12** has two electrophilic centres,[^10b^, ^11^] the accumulating product is likely a mixture of two regioisomers (Figure 3). The MeI‐containing reaction converted all peptide to the methyl ester in the first hr (**11**, [C_104_H_167_N_25_O_32_]^2+^ calcd: m/z 1139.612, found: 1139.604, Figure S22), but formation of the conjugate (**15**) occurred much more slowly. Fitting the time dependent concentrations of the different peptide species to a sequential two‐step reaction (see Supporting Information for details) shows that the rate of intermolecular conjugation with methoxyamine is similar in both reactions (*k*_2_, Figure 3), consistent with the idea that methoxyamine reacts with the same electrophilic succinimide intermediate (**12**). Formation of this obligatory intermediate, on the other hand, occurs 10‐fold faster with FMeI (*k*_1_) than with MeI (*k*_1_′) as the conjugating agent.

F‐methylation was also effective in labelling this model peptide with hydroxylamine‐derivatized biotin in a one‐pot reaction (**16**, [C_121_H_198_N_29_O_37_S]^3+^ calcd: m/z 894.141, found: 894.134, Figure S23). PIMT‐mediated conjugation to biotin has been proposed as a strategy for enriching and identifying low‐abundance isoAsp‐containing peptides and proteins in proteome samples. However, no reports of such conjugates being synthesized have been published.^[15a]^

A second type of SAM‐dependent MTs with potential applications in protein ligation may be the carboxylate MT LahS_B_ from *Lachnospiraceae bacterium* (PDB: 6UAK).^[18]^ This enzyme methylates the C‐terminal Val, Ile or Met residues of several translated precursors of peptide natural products (LahA2–A5). Unlike other protein carboxylate MTs, such as the eukaryotic leucine carboxyl MT or the lanthipeptide maturing enzyme OlvSA which require their substrates to adopt a stable three‐dimensional fold for recognition,^[19]^ LahS_B_ appears to recognize unmodified substrates by just the last 15, or possibly fewer residues.

Based on this report, we designed an expression plasmid coding for a green fluorescent protein (GFP, PDB: 2B3P)^[20]^ with a 15‐residue C‐terminal tag mimicking the terminus of the precursor peptide LahA2 (sequence: DGDEVDYSLFAATAM, GFP‐tag‐1). Incubation at pH 8.0 in the presence of 1 mM SAM, 10 μM LahS_B_ for 4 h at 25°C converted GFP‐tag‐1 (20 μM) completely to methylated GFP‐tag‐1 (**17**) as inferred by HR‐ESI‐MS (**17**, calcd: m/z 30509.3, found: 30509, Figure S24). A reaction containing 1 mM FMeI, 20 μM SAH, 10 μM LahS_B_, 10 μM HMT and 2 mM cysteine converted more than 90 % of GFP‐tag‐1 (20 μM) to the cysteine adduct **19** as inferred by relative signal intensities of the corresponding HR‐ESI‐MS spectrum (calcd: m/z 30598.8, found: 30598, **A**, Figure 4 and S25). Increasing or decreasing the reaction pH (6.5–8.5) reduced this yield significantly, presumably due to an increased rate of ester hydrolysis or reduced concentration of anionic Cys (Figures S26–S29).

**Figure 4:**
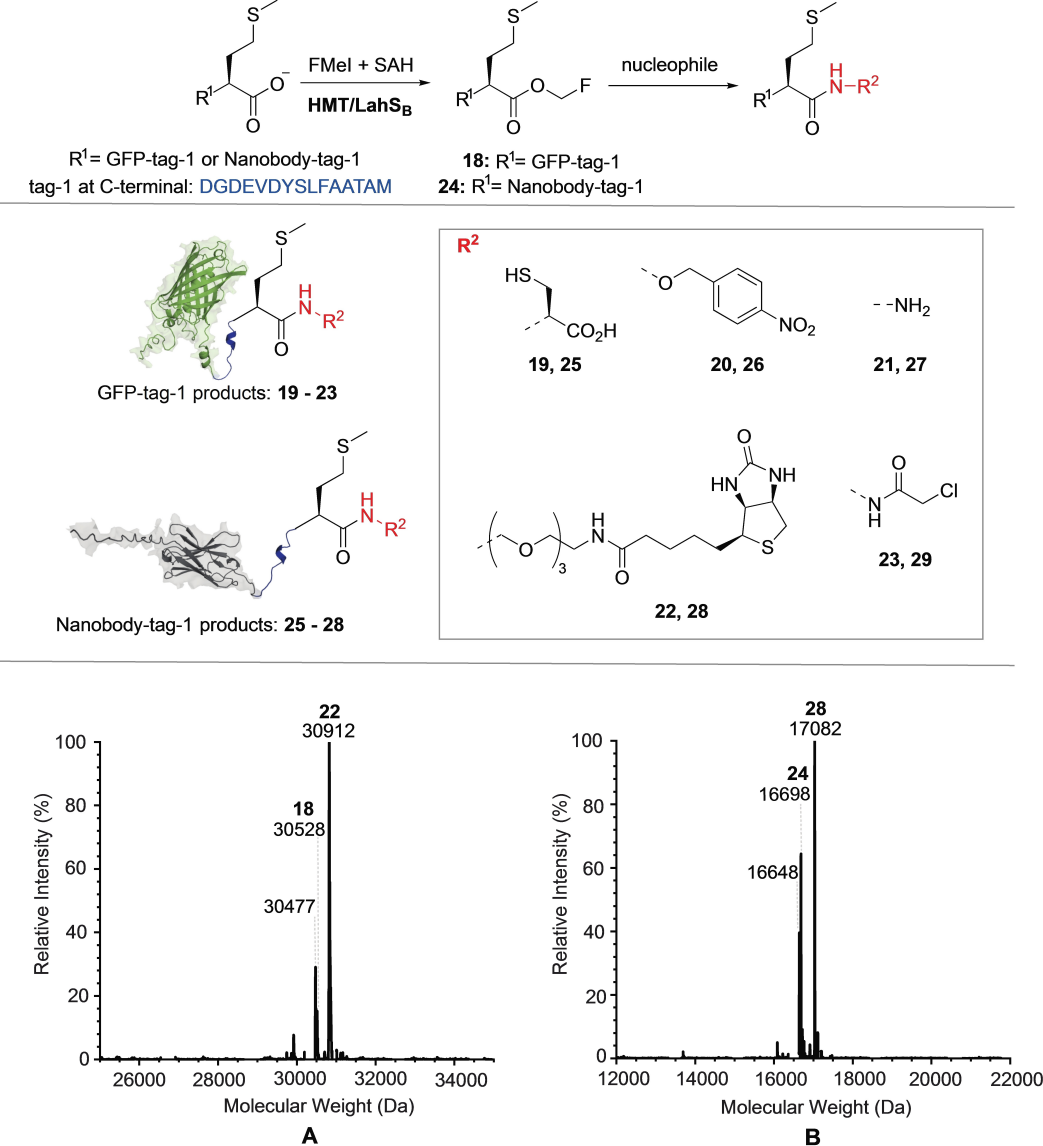
Protein conjugation to different nucleophiles via F‐methyl ester. **Top**: LahS_B_ catalyzed F‐methylation of GFP‐tag‐1 and nanobody‐tag‐1 followed by conjugation with small nucleophiles. **Middle** Protein conjugates produced by MT‐mediated activation. **Bottom**: **A)** Mass spectrum of the reaction after 48 hrs of incubating GFP‐tag‐1 with biotin oxyamine, giving the mass of product **22** (calcd: m/z 30912.3, found: 30912). Additional signals indicate the presence of the F‐methyl ester **18** (calcd: m/z 30527.8, found: 30528) and the anhydride of GFP‐tag‐1 (GFP‐tag‐1‐H_2_O, calcd: m/z 30477.4, found: 30477). See reaction details in Figure S33. **B)** Mass spectrum of the reaction mixture after 48 hrs of incubating nanobody‐tag‐1 with biotin oxyamine, giving the mass of **28** (calcd: m/z 17083.6, found: 17082). Additional signals indicate the presence of the F‐methyl ester **24** (calcd: m/z 16699.9, found: 16698) and the anhydride nanobody‐tag‐1‐H_2_O (calcd: m/z 16649.2, found: 16648). See reaction details in Figure S40.

Recombinant versions of GFP with different lanthipeptide‐inspired C‐termini (tag‐2: GSGFLGSAIVAASSAGAV; tag‐3: GSGDGDEVDYSLFAATAI) were also modified by LahS_B_, but the resulting F‐methyl esters (GFP‐tag‐2: calcd: m/z 30243.4, found: 30243.1; GFP‐tag‐3: calcd: m/z 30509.6, found: 30508.8; Figure S30 and S31) ligated much less efficiently to Cys (<30 %). We suspect that the lower conjugation efficiencies are related to steric hinderance by the β‐branched side chains of the C‐terminal Val and Ile residues.^[21]^ F‐methylation of GFP‐tag‐1 in the presence of a hydroxylamine‐derivatized dye (4‐nitrobenzylhydroxylamine, 20 mM), hydrazine (20 mM) or a hydroxylamine derivatized biotin (20 mM) produced more than 70 % of the corresponding conjugates (**20**–**22**, Figure 4, Figure S32–S44). Conjugate **23** was obtained by incubation of the GFP‐hydrazide **21** with chloroacetic anhydride (1–50 mM) at pH 3.0, in adaptation of a recently published protocol for the installation of C‐terminal chloride electrophiles (Figure S44).^[22]^ In the absence of an efficient external nucleophile, F‐methylation of GFP‐tag‐1 resulted in transient accumulation of the F‐methyl ester (**18**), which, after 19 hrs, completely transformed into a species that is 18 Da lighter than unmodified GFP‐tag‐1 (GFP‐tag‐1‐H_2_O: calcd: m/z 30477.4, found: 30478, Figure S36), suggesting that the F‐methylation protein has the capacity to slowly undergo intramolecular condensation. The same species (GFP‐tag‐1‐H_2_O) also forms in some of the conjugation reactions described above as a minor side product.

The same methodology was used to conjugate small molecules to a nanobody (PDB: 3OGO)^[23]^ with a C‐terminal tag‐1 (**24**–**28**, Figure 4 and S37–S42). These results suggest that MT‐mediated C‐terminal protein labeling may be an efficient and easy to implement alternative to transpeptidase‐mediated ligation technologies.^[24]^ One potential advantage of the present approach over these established methodologies is that conjugation to the F‐methyl esters does not require catalysis and therefore tolerate nucleophiles that are not accepted by transpeptidases.

Finally, we examined as to whether the F‐methylated C‐terminus of GFP‐tag‐1 could also react with the N‐terminal Cys residue of another protein (Figure 5). To this end we incubated 0.3 mM GFP‐tag‐1 with 0.6 mM of a nanobody variant that features an N‐terminal Cys residue (nanobody‐C16) together with HMT (20 μM), LahS_B_ (20 μM), SAH (40 μM), a total of 8 mM FMeI in 50 mM phosphate buffer at pH 8.0 at 15 °C. After 48 h the reaction mixture was analyzed by SDS‐PAGE and HR‐ESI‐MS (Figure 5 and S43). Both analyses show that more that 50 % of GFP‐tag‐1 has ligated to the nanobody (GFP‐tag‐1‐nanobody‐C16: calcd: m/z 41782.1, found: 41782). The remaining GFP‐tag‐1 was converted to the −18 Da anhydride of GFP‐tag‐1 (GFP‐tag‐1‐H_2_O) as a result of intramolecular condensation. This anhydride likely emerges from intramolecular condensation between the C‐terminal F‐methyl ester and a nucleophilic side chain of the protein. Indeed, incubation of GFP‐tag‐1‐H_2_O with 2 M hydroxylamine at pH 8.0 and 25 °C for 200 min produced a mixture of GFP‐tag‐1 (by hydrolysis) and GFP‐tag‐1‐hydroxamate (by hydroxylaminolysis, Figure S44).

**Figure 5:**
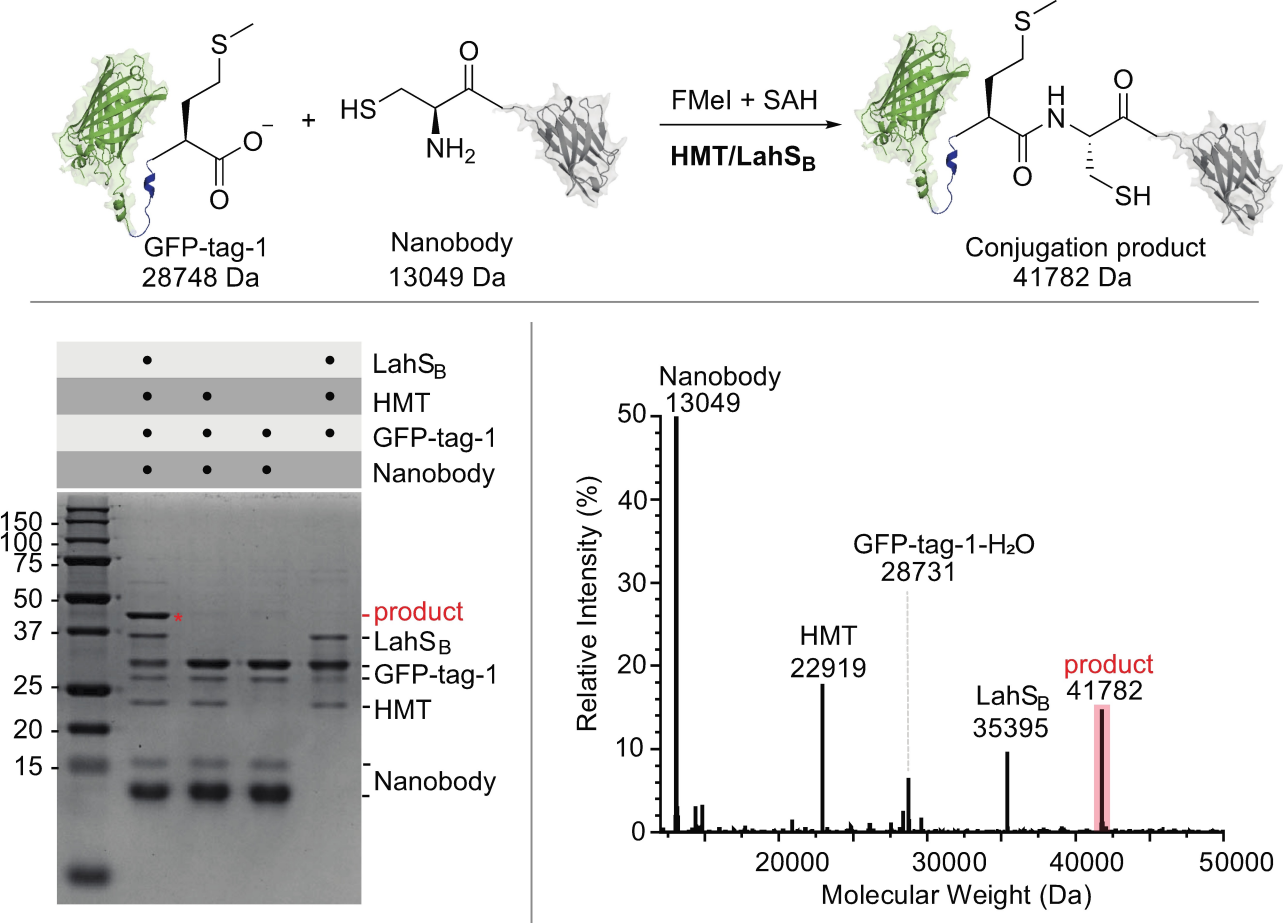
**Top**: Methyltransferase mediated ligation between GFP‐tag‐1 (calcd: m/z 28750.3) and nanobody‐C16 (calcd: m/z 13050.5, found: 13049). **Bottom left**: SDS‐PAGE analysis of the ligation reaction and the control experiments after 48 hrs. A new band corresponding to the protein conjugation product is marked with a red asterisk. **Lane 1**: full ligation reaction; **Lane 2**: control reaction lacking LahS_B_ (calcd: m/z 35396.4, found: 35395). **Lane 3**: control reaction lacking HMT (calcd: m/z 22919.7, found: 22919) and LahS_B_. **Lane 4**: control reaction lacking nanobody‐C16. **Bottom right**: Mass spectrum of the ligation reaction after 48 hrs. Comparison of the signal intensities of the ligation product (calcd: m/z 41782.1, found: 41782) and GFP‐tag‐1‐H_2_O (calcd: m/z 28732.3, found: 28731) suggest that approximately 60 % of GFP‐tag‐1 was ligated to nanobody‐C16.

Chemoselective conjugation of a protein with an activated C‐terminus to a protein with an N‐terminal Cys to form an eupeptide bond has been termed native chemical ligation.^[25]^ One of the most important discoveries in chemical biology, this methodology has enabled the assembly of chemically defined large proteins from synthetic peptides and recombinant protein fragments.^[26]^ Although there are established protocols for the production of peptides with activated C‐termini,^[27]^ we anticipate that MT‐mediated in situ activation of recombinant proteins can open up new options for protein synthesis, not the least because this approach is simple to implement and requires only off‐the‐shelf chemical reagents. Combining protein F‐methylation with ligation auxiliaries and selenium‐assisted ligation,^[28]^ provide promising options to further improve the observed conjugation efficiency and possibly suppress unwanted side products.

In conclusion, MT‐mediated F‐methylation of carboxylic acids produces reactive F‐methyl esters that readily condense with *N*‐ and *S*‐nucleophiles under physiological conditions. We have illustrated possible applications of this reaction in the synthesis of small thioesters, hydroxamates, and amides, as a tool for isoAsp‐specific and C‐terminal protein conjugation, and for protein synthesis by native chemical ligation. The simplicity and broad substrate scope of MT‐mediated ligation makes this approach amenable to further improvement and adaptation. As an exciting example we note the recent discovery that (formal) substitution of the sulfur atom in F‐SAM to tellurium generates a more stable reagent for enzyme‐catalyzed F‐methylation.^[29]^

## Conflict of interest

The authors declare no conflict of interest.

## Supporting information

As a service to our authors and readers, this journal provides supporting information supplied by the authors. Such materials are peer reviewed and may be re‐organized for online delivery, but are not copy‐edited or typeset. Technical support issues arising from supporting information (other than missing files) should be addressed to the authors.

Supporting Information

## Data Availability

The data that support the findings of this study are available in the supplementary material of this article.

## References

[1a] A. El-Faham , F. Albericio , Chem. Rev. 2011, 111, 6557–6602; 21866984 10.1021/cr100048w

[1b] M. T. Sabatini , L. T. Boulton , H. F. Sneddon , T. D. Sheppard , Nat. Catal. 2019, 2, 10–17;

[1c] Y. Tan , H. Wu , T. Wei , X. Li , J. Am. Chem. Soc. 2020, 142, 20288–20298; 10.1021/jacs.0c09664 33211477

[1d] R. M. de Figueiredo , J. S. Suppo , J. M. Campagne , Chem. Rev. 2016, 116, 12029–12122; 27673596 10.1021/acs.chemrev.6b00237

[1e] C. Bednarek , I. Wehl , N. Jung , U. Schepers , S. Bräse , Chem. Rev. 2020, 120, 4301–4354; 32356973 10.1021/acs.chemrev.9b00665

[1f] V. R. Pattabiraman , J. W. Bode , Nature 2011, 480, 471–479; 22193101 10.1038/nature10702

[1g] C. L. Allen , J. M. J. Williams , Chem. Soc. Rev. 2011, 40, 3405–34015. 21416075 10.1039/c0cs00196a

[2a] M. Lubberink , W. Finnigan , S. L. Flitsch , Green Chem. 2023, 25, 2958–2970;

[2b] A. Goswami , S. G. Van Lanen , Mol. BioSyst. 2015, 11, 338–353; 25418915 10.1039/c4mb00627e PMC4304603

[2c] B. M. Dorr , D. E. Fuerst , Curr. Opin. Chem. Biol. 2018, 43, 127–133; 29414531 10.1016/j.cbpa.2018.01.008

[2d] M. Winn , S. M. Richardson , D. J. Campopiano , J. Micklefield , Curr. Opin. Chem. Biol. 2020, 55, 77–85; 32058241 10.1016/j.cbpa.2019.12.004

[2e] H. Müller , H. Terholsen , S. P. Godehard , C. P. S. Badenhorst , U. T. Bornscheuer , ACS Catal. 2021, 11, 14906–14915;

[2f] A. Stanišić , K. H , ChemBioChem 2019, 20, 1347–1356. 30629787 10.1002/cbic.201800750

[3a] M. Winn , M. Rowlinson , F. Wang , L. Bering , D. Francis , C. Levy , J. Micklefield , Nature 2021, 593, 391–398; 34012085 10.1038/s41586-021-03447-w

[3b] T. Wang , Y. F. Zhang , L. X. Ning , Y. F. Wang , X. H. Liu , R. Li , X. E. Chen , Enzyme Microb. Technol. 2020, 136, 109537. 32331719 10.1016/j.enzmictec.2020.109537

[4a] M. Lubberink , W. Finnigan , C. Schnepel , C. R. Baldwin , N. J. Turner , S. L. Flitsch , Angew. Chem. Int. Ed. 2022, 61, e202205054; 10.1002/anie.202205054 PMC9401052 35595679

[4b] C. Schnepel , L. Rodríguez Pérez , Y. Yu , A. Angelastro , R. S. Heath , M. Lubberink , F. Falcioni , K. Mulholland , M. A. Hayes , N. J. Turner , S. L. Flitsch , Nat. Catal. 2023, 6, 89–99;

[4c] M. R. Petchey , B. Rowlinson , R. C. Lloyd , I. J. S. Fairlamb , G. Grogan , ACS Catal. 2020, 10, 4659–4663. 32337091 10.1021/acscatal.0c00929 PMC7171872

[5] M. Funabashi , Z. Yang , K. Nonaka , M. Hosobuchi , Y. Fujita , T. Shibata , X. Chi , S. G. Van Lanen , Nat. Chem. Biol. 2010, 6, 581–586. 20562876 10.1038/nchembio.393

[6] L. C. Ward , H. V. McCue , A. J. Carnell , ChemCatChem 2021, 13, 121–128.

[7] J. H. Peng , C. Liao , C. Bauer , S. F. P , Angew. Chem. Int. Ed. 2021, 60, 27178–27183. 10.1002/anie.202108802 34597444

[8] N. Zhao , J. L. Ferrer , J. Ross , J. Guan , Y. Yang , E. Pichersky , J. P. Noel , F. Chen , Plant Physiol. 2008, 146, 455–467. 18162595 10.1104/pp.107.110049 PMC2245846

[9] H. M. Burke , L. McSweeney , E. M. Scanlan , Nat. Commun. 2017, 8, 15655. 28537277 10.1038/ncomms15655 PMC5458133

[10a] T. Krüger , S. Weiland , G. Falck , M. Gerlach , M. Boschanski , S. Alam , K. M. Müller , T. Dierks , N. Sewald , Angew. Chem. Int. Ed.. 2018, 57, 7245–7249; 10.1002/anie.201803183 29579347

[10b] K. J. Reissner , D. W. Aswad , Cell. Mol. Life Sci. 2003, 60, 1281–1295; 12943218 10.1007/s00018-003-2287-5 PMC11138701

[10c] P. N. McFadden , S. Clarke , Proc. Natl. Acad. Sci. USA 1987, 84, 2595–2599. 3472227 10.1073/pnas.84.9.2595 PMC304704

[11a] T. Geiger , S. Clarke , J. Biol. Chem. 1987, 262, 785–794; 3805008

[11b] S. Capasso , A. J. Kirby , S. Salvadori , F. Sica , A. Zagari , J. Chem. Soc. Perkin Trans. 2 1995, 437–442.

[12] L. Ogé , G. Bourdais , J. Bove , B. Collet , B. Godin , F. Granier , J. P. Boutin , D. Job , M. Jullien , P. Grappin , Plant Cell 2008, 20, 3022–3037. 19011119 10.1105/tpc.108.058479 PMC2613667

[13] J. E. Visick , H. Cai , S. Clarke , J. Bacteriol. 1998, 180, 2623–2629. 9573145 10.1128/jb.180.10.2623-2629.1998 PMC107212

[14a] W. M. Hicks , M. V. Kotlajich , J. E. Visick Microbiology 2005, 151, 2151–2158; 16000706 10.1099/mic.0.27835-0

[14b] L. Yin , C. S. Harwood , J. Biol. Chem. 2019, 294, 2854–2861. 30578298 10.1074/jbc.RA118.006546 PMC6393607

[15a] J. F. Alfaro , L. A. Gillies , H. G. Sun , S. Dai , T. Zang , J. J. Klaene , B. J. Kim , J. D. Lowenson , S. G. Clarke , B. L. Karger , Z. S. Zhou , Anal. Chem. 2008, 80, 3882–3889; 18419136 10.1021/ac800251q

[15b] M. M. Müller , Biochemistry 2018, 57, 177–185; 29064683 10.1021/acs.biochem.7b00861 PMC5770884

[15c] J. J. Klaene , W. Ni , J. F. Alfaro , Z. S. Zhou , J. Pharm. Sci. 2014, 103, 3033–3042. 25043726 10.1002/jps.24074 PMC4175021

[16] J. W. Silzel , T. R. Lambeth , R. R. Julian , J. Am. Soc. Mass Spectrom. 2022, 33, 548–556. 35113558 10.1021/jasms.1c00355 PMC9930442

[17] P. G. Kabadi , P. K. Sankaran , D. V. Palanivelu , L. Adhikary , Khedkar , A. Chatterjee , J. Am. Soc. Mass Spectrom. 2014, 27, 1677–1685. 10.1007/s13361-016-1447-4 27488315

[18] L. Huo , X. Zhao , J. Z. Acedo , P. Estrada , S. K. Nair , W. A. van der Donk , ChemBioChem 2020, 21, 190–199. 31532570 10.1002/cbic.201900483 PMC6980331

[19a] J. Z. Acedo , I. R. Bothwell , L. An , A. Trouth , C. Frazier , W. A. van der Donk , J. Am. Chem. Soc. 2019, 141, 16790–16801; 31568727 10.1021/jacs.9b07396 PMC6812601

[19b] N. Leulliot , S. Quevillon-Cheruel , I. Sorel , I. Li de La Sierra-Gallay , B. Collinet , M. Graille , K. Blondeau , N. Bettache , A. Poupon , J. Janin , H. van Tilbeurgh , J. Biol. Chem. 2004, 279, 8351–8358; 14660564 10.1074/jbc.M311484200

[19c] V. Stanevich , L. Jiang , K. A. Satyshur , Y. Li , P. D. Jeffrey , Z. Li , P. Menden , M. F. Semmelhack , Y. Xing , Mol. Cell 2011, 41, 331–342. 21292165 10.1016/j.molcel.2010.12.030 PMC3060061

[20] J. D. Pédelacq , S. Cabantous , T. Tran , T. C. Terwilliger , G. S. Waldo , Nat. Biotechnol. 2006, 24, 79–88. 16369541 10.1038/nbt1172

[21] V. Diemer , O. Firstova , V. Agouridas , O. Melnyk , Chemistry 2022, 28, e202104229. 35048443 10.1002/chem.202104229

[22] J. Farnung , K. A. Tolmachova , J. W. Bode , Chem. Sci. 2023, 14, 121–129. 10.1039/d2sc04279g PMC9769091 36605735

[23] M. H. Kubala , O. Kovtun , K. Alexandrov , B. M. Collins , Protein Sci. 2010, 19, 2389–2401. 20945358 10.1002/pro.519 PMC3009406

[24a] F. B. H. Rehm , T. J. Tyler , K. Yap , S. J. De Veer , D. J. Craik , T. Durek , J. Am. Chem. Soc. 2021, 143, 19498–19504; 34761936 10.1021/jacs.1c08976

[24b] R. J. Taylor , M. B. Geeson , T. Journeaux , G. J. L. Bernardes , J. Am. Chem. Soc. 2022, 32, 14404–14419 ; 10.1021/jacs.2c00129 PMC9389620 35912579

[24c] H. E. Morgan , W. B. Turnbull , M. E. Webb , Chem. Soc. Rev. 2022, 51, 4121–4145; 35510539 10.1039/d0cs01148g PMC9126251

[24d] M. Schmidt , A. Toplak , P. J. Quaedflieg , T. Nuijens , Curr. Opin. Chem. Biol. 2017, 38, 1–7; 28229906 10.1016/j.cbpa.2017.01.017

[24e] R. Pihl , Q. F. Zheng , Y. David , Nat. Chem. Rev. 2023, 7, 234–255; 10.1038/s41570-023-00468-z PMC10659114 37117416

[24f] G. K. T. Nguyen , S. Wang , Y. Qiu , X. Hemu , Y. Lian , J. P. Tam , Nat. Chem. Biol. 2014, 10, 732–738; 25038786 10.1038/nchembio.1586

[24g] A. Haim , S. Neubacher , T. N. Grossmann , ChemBioChem 2021, 22, 2672–2679. 34060202 10.1002/cbic.202100111 PMC8453710

[25] P. E. Dawson , T. W. Muir , I. Clark-Lewis , S. B. Kent , Science 1994, 266, 776–779. 7973629 10.1126/science.7973629

[26a] A. C. Conibear , E. E. Watson , R. J. Payne , C. F. W. Becker , Chem. Soc. Rev. 2018, 47, 9046–9068; 30418441 10.1039/c8cs00573g

[26b] R. E. Thompson , T. W. Muir , Chem. Rev. 2020, 120, 3051–3126; 31774265 10.1021/acs.chemrev.9b00450 PMC7101271

[26c] V. Agouridas , O. El Mahdi , V. Diemer , M. Cargoët , J. M. Monbaliu , O. Melnyk , Chem. Rev. 2019, 119, 7328–7443; 31050890 10.1021/acs.chemrev.8b00712

[26d] B. Yan , W. Shi , L. Ye , L. Liu , Curr. Opin. Chem. Biol. 2018, 46, 33–40; 29654943 10.1016/j.cbpa.2018.03.018

[26e] C. P. Hackenberger , D. Schwarzer , Angew. Chem. Int. Ed. 2008, 47, 10030–10074. 10.1002/anie.200801313 19072788

[27a] T. W. Muir , D. Sondhi , P. A. Cole , Proc. Natl. Acad. Sci. USA 1998, 95, 6705–6710; 9618476 10.1073/pnas.95.12.6705 PMC22605

[27b] H. J. Reich , R. J. Hondal , ACS Chem. Biol. 2016, 11, 821–841. 26949981 10.1021/acschembio.6b00031

[28a] C. C. Hanna , J. Kriegesmann , L. J. Dowman , C. F. W. Becker , R. J. Payne , Angew. Chem. Int. Ed. 2022, 61, e202111266; 10.1002/anie.202111266 PMC9303669 34611966

[28b] N. J. Mitchell , L. R. Malins , X. Liu , R. E. Thompson , B. Chan , L. Radom , R. J. Payne , J. Am. Chem. Soc. 2015, 137, 14011–14014; 26487084 10.1021/jacs.5b07237

[28c] R. J. Hondal , B. L. Nilsson , R. T. Raines , J. Am. Chem. Soc. 2001, 123, 5140–5141; 11457362 10.1021/ja005885t

[28d] R. Quaderer , D. Hilvert , Helv. Chim. Acta 2001, 84, 1197–1206;

[28e] O. Fuchs , S. Trunschke , H. Hanebrink , M. Reimann , O. Seitz , Angew. Chem. Int. Ed. 2021, 60, 19483–19490; 10.1002/anie.202107158 PMC8457107 34165893

[28f] J. M. Bondalapati , A. Brik , Nat. Chem. 2016, 8, 407–418; 27102674 10.1038/nchem.2476

[28g] R. J. Giesler , P. W. Erickson , M. S. Kay , Curr. Opin. Chem. Biol. 2020, 58, 37–44; 32745915 10.1016/j.cbpa.2020.04.003 PMC8106950

[28h] R. Mousa , R. Notis Dardashti , N. Metanis , Angew. Chem. Int. Ed. 2017, 56, 15818–15827. 10.1002/anie.201706876 28857389

[29] S. S. Neti , B. Wang , D. F. Iwig , E. L. Onderko , S. J. Booker , ACS Cent. Sci. 2023, 9, 905–914. 37252363 10.1021/acscentsci.2c01385 PMC10214534

